# Replication of epistatic DNA loci in two case-control GWAS studies using OPE algorithm

**DOI:** 10.1186/1471-2105-12-S11-A5

**Published:** 2011-11-21

**Authors:** Benjamin Goudey, Qiao Wang, Dave Rawlinson, Armita Zarnegar, Eder Kikianty, John Markham, Geoff Macintyre, Gad Abraham, Linda Stern, Michael Inouye, Izhak Haviv, Adam Kowalczyk

**Affiliations:** 1 Department of Software Engineering and Computer Science, The University of Melbourne, Parkville, Victoria 3010, Australia; 2 National ICT Australia (NICTA) Victoria Research Laboratories, The University of Melbourne, Parkville, Victoria 3010, Australia; 3 The Walter and Eliza Hall Institute of Medical Research, Parkville, Victoria 3050, Australia; 4 Baker IDI Heart and Diabetes Institute, Melbourne, Victoria 3004, Australia; 5 Department of Medical Biology, University of Melbourne, Parkville, Victoria 3010, Australia

## Background

One of the limiting factors of current genome-wide association studies (GWAS) is the inability of current methods to comprehensively examine SNP interactions for a reasonable sized dataset. It is hypothesised that this limitation is one of the reasons that GWAS studies have not been able to have a greater impact [1,2]. Many current methods for handling interactions are computationally expensive and do not scale to entire studies. Those methods that do scale often achieve this by pruning their datasets in some manner. This is commonly done by considering only those SNPs that show strong marginal effects, despite the fact that a strongly interacting pair may consist of SNPs with low effects individually.

## Material and methods

In this presentation, we validate the robustness of a novel algorithm known as Optimal Pairwise Epistasis (OPE) for exhaustively examining all pairwise interactions in GWAS data. This method is based on the systematic evaluation of “binary genotype pairs” (BG-pairs), i.e. the pairs of complementary binary classification of genotype calls for an individual SNP, or a pair of SNPs. We can quantify the discrimination potential of BG-pairs using a family of statistics based on odds ratios.

## Results and conclusion

The approach is computationally efficient: the dataset reported here as Study 1 (consisting of ~310K SNPs and 2200 samples [3]) takes 12 hour to process on a single CPU (compared to 149 hours of the recent BOOST algorithm [4]). The method can be highly parallelised with a recent GPU implementation reducing this processing time to less than 15 minutes.

**Figure 1:**
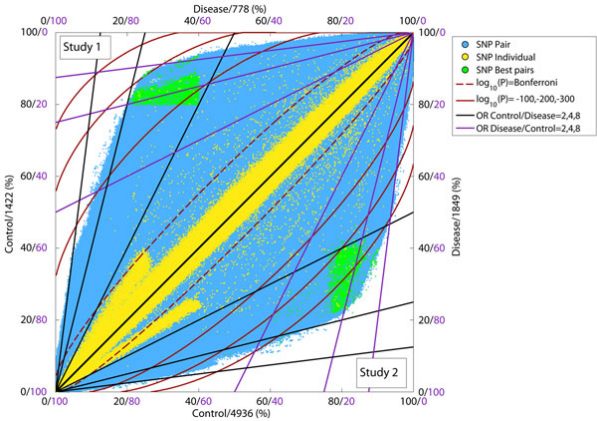
Plot of Binary Genotypes (BG-pairs) for pairs/individual SNPs in 2 independent Celiac GWAS studies.

We have tested our approach over 2 independent GWAS studies of Celiac disease: the first (Study 1 mentioned above, [3]) with 778/1422 and the second (Study 2, [5]) with 1849/4936 of case/control samples, respectively. Each point in the figure 1 below shows the observed frequency of the BG carriers for the case and control subpopulations: in blue for a pair of SNPs or in yellow for an individual SNP. Every BG-pair can be evaluated with respect to the two sets of axes labels: purple labels for the protective BG and black labels for the risk BG. The resulting figure shows both studies related by symmetry in the main diagonal and indicates replication of results across studies. We emphasise the replicability of our approach by showing in green the same subset of SNP pairs in both studies. We also show in red contours for p-values and plot in black / purple solid diagonal lines to indicate different odds ratios.
